# International migrant workers, heat exposure, and climate change: a systematic review of health risks and protective interventions

**DOI:** 10.1186/s44263-025-00224-z

**Published:** 2025-12-01

**Authors:** Lara Van der Horst, Oumnia Bouaddi, Sarah Williams, Meg Jago, Karen Lau, Ben Furber, Amirah Zafirah Zaini, Engy Mohamed El-Ghitany, Tharani Loganathan, Andreas D. Flouris, Davide J. Testa, Cathy Zimmerman, Sally Hargreaves, Consortium for Migrant Worker Health

**Affiliations:** 1 The Migrant Health Research Group, Institute for Infection and Immunity, University of London, City St George’s, London, United Kingdom; 2 Mohammed VI International School of Public Health, Mohammed VI University of Sciences and Health, Casablanca, Morocco; 3 Department of Public Health and Clinical Research, Mohammed VI Center for Research and Innovation, Rabat, Morocco; 4 Barcelona Institute of Global Health, Barcelona, Spain; 5 Department of Global Health and Development, Faculty of Public Health and Policy, London School of Hygiene and Tropical Medicine, London, UK; 6 Centre for Epidemiology and Evidence-Based Practice, Department of Social and Preventive Medicine, Faculty of Medicine, Universiti Malaya, Kuala Lumpur, Malaysia; 7 High Institute of Public Health, University of Alexandria, Alexandria, Egypt; 8 FAME Laboratory, Department of Physical Education and Sport Science, University of Thessaly, Trikala, Greece

**Keywords:** International migrants, Occupational Health, Migrant workers, Heat exposure, Climate change

## Abstract

**Background:**

International migrant workers, representing 170 million people globally, often face hazardous working conditions, including extreme heat exposure. These increase their risk of occupational heat strain, exacerbated by poor and exploitative working conditions. This systematic review aims to identify the health risks associated with occupational heat exposure among international migrant workers and document protective interventions and measures being used globally, to inform policies that protect this vulnerable population.

**Methods:**

We searched four electronic databases (Medline, Embase, Ovid Global Health and PsychINFO) for primary research studies (January 2014–April 2024) on international migrant workers experiencing adverse health outcomes alongside high working temperatures. Records were screened, and data were extracted by two independent reviewers. Assessment of study quality was done using Joanna-Briggs Institute checklists. Results were synthesised narratively and reported following PRISMA 2020 guidelines.

**Results:**

Of the 646 records screened, 19 studies involving 2293 migrant workers across six countries were included in the analysis, most of which were conducted in high-income countries (*n = *14, 74%), mainly the United States of America (USA). At-risk workers, with ages ranging 10-90 years, were employed in construction (48%) and agriculture (42%), and originated from 14 countries, predominantly India, Mexico, and Nepal. Studies reported workers affected by heat-related illnesses (*n = *12 studies), dehydration (*n *= 5), kidney disease (*n = *2), and poor skin health (*n = *2). Workers most commonly suffered from symptoms of headaches (*n = *83 workers), muscle cramps (*n = *53), and heavy sweating (*n = *44), with other issues including poor mental health, infertility, and risk to pregnancy interventions focused on water, rest, shade, skin protection, and education, but evaluations were limited and some measures failed to address heat exposure effectively.

**Conclusions:**

Occupational heat exposure poses significant health risks for international migrant workers. Where interventions exist, barriers to effectiveness remain, with little evidence from low- and middle- income countries. Amid rising global temperatures, a greater focus is needed on improved worker education, worker-tailored and co-designed interventions, updated guidelines, and increased healthcare accessibility.

**Systematic review registration:**

PROSPERO CRD42024519547.

**Supplementary Information:**

The online version contains supplementary material available at 10.1186/s44263-025-00224-z.

## Background

International migrant workers are defined as individuals who are to be engaged, are engaged or have been engaged in a remunerated activity in a state of which they are not nationals [1]. They make up 170 million of the world’s population and a significant portion of the labour workforce [2]. The top recorded destinations of work are Europe, North America, and the Middle East, with low and middle-income countries (LMICs) receiving remittances valued at billions of United States Dollars (USD) each year [2, 3]. Migrant workers often work in irregular settings with limited legal protection making them more vulnerable to occupational health risks [4]. Workers are often paid less than their non-migrant counterparts and are subjected to worse working conditions including more strenuous work, less flexible work schedules and exploitative financial arrangements [5–7]. In addition, they are more likely to experience occupational health injuries and less likely to use health services than non-migrant workers [8]. For instance, in a recent global meta-analysis among 7260 international migrant workers, the pooled prevalence of having at least one occupational morbidity was 47% (95% confidence interval (CI) 29–64%; *I*^2 ^= 99·70%) [9]. As a result of these trends, migrants are commonly referred to as working in 3D jobs, a term used to describe jobs that may be a combination of dirty, demeaning, demanding, dangerous, and difficult [10, 11].

Globally, it is estimated that 33.8% of international migrant workers are employed in farming, manufacturing, mining and quarrying, and construction [12]. Workers in these sectors typically perform their duties outdoors, increasing their exposure to environmental heat. Physiological heat stress causes heat strain, presenting as symptoms of excessive sweating, and feeling hot and thirsty. This, in turn, increases the risk of subsequent heat-related illnesses (HRIs). HRI encompasses a group of acute conditions brought about by heat stress. These conditions include heat rash or heat cramps, the onset of heat syncope or heat exhaustion, and in more serious cases rhabdomyolysis or heat stroke. In occupational settings, where heat exposure may be combined with heavy physical labour, resultant adverse health effects may be collectively known as OHS. OHS has been found to include heat stroke, heat cramps, and heat exhaustion, and 15% of cases involve kidney disease or acute kidney injury (AKI) [13]. Where legislation exists to protect workers they may still find themselves at risk. For example, the Gulf countries (Bahrain, Kuwait, Oman, Qatar, Saudi Arabia and the United Arab Emirates) have implemented bans on midday working and measures to ensure the availability of drinking water, shade, and personal protective equipment (PPE) [14]. However, an assessment of the ban on midday work showed that it is not entirely effective on its own and should be combined with the provision of shaded areas, access to cool water and rehydration salts, and medical checks [15].

In the Gulf Country of Kuwait, one study found that migrant workers experience a three-fold higher risk of mortality from high temperatures [16]. Worse heat-health risk profiles in migrant workers occur as a result of personal factors like a smaller body size, preferences for wearing more clothing—in some cases for cultural and religious reasons, and working more intensely with fewer rest breaks [17]. Furthermore, greater vulnerability to occupational heat strain also results from language and cultural barriers, further reducing access to resources for the prevention and treatment of heat-related illnesses [18, 19].

There are multiple previous reviews exploring the effects of occupational heat exposure on workers’ health and productivity, finding that such exposure leads to OHS, more occupational injuries, increased risk of vector-borne disease, and reduced productivity [13, 18, 20–22]. However, evidence is limited in terms of international migrant workers. El Khayat et al. identify migrant workers as a vulnerable group calling for increased research into their experiences, specifically [18]. Reviews investigating effects on migrant workers are fewer and confirm migrants’ vulnerability to occupational injuries [9]; one concludes that there is a high burden of heat-related illness amongst this group [23]. To our knowledge, this is the first review to focus specifically on international migrant workers with an additional analysis on protective interventions to inform improvements in preventative efforts to protect this group [23].

The United Nations (UN) Sustainable Development Goals has acknowledged the protection of migrant health as a global priority [24]. The extension of the Global Action Plan Promoting the Health and Wellbeing of Refugees and Migrants, also demonstrates a similar intention [25]. The unique risk factors for occupational heat exposure among migrant workers, and their limited access to protective resources call for strengthening health and labour policies targeted to this vulnerable group for these priorities to be met. Gaining a deeper understanding of the risks facing international migrants working in hot conditions is the first step to guiding the development of such policy.

The primary objective of this systematic review is to identify the direct and indirect health risks among migrant workers following occupational heat exposure. The secondary objective is to identify current interventions and strategies to mitigate adverse health effects following occupational heat exposure in migrant workers.

## Methods

This systematic review is reported according to the Preferred Reporting items for Systematic Review and Meta-Analysis (PRISMA) 2020 guidelines [26] (see Additional file 1).

### Search strategy

We searched four electronic databases including MEDLINE (Ovid), Global Health (Ovid), PsychINFO (Ovid), Embase (Ovid) and the Cochrane library for studies reporting health outcomes in migrant workers following occupational heat exposure published between 01/01/2014 and 31/04/2024. This period was selected to represent a time within which global warming has been attributed to anthropogenic climate change, with 2015 identified as an initial year in which labour productivity was significantly reduced due to the impact of heat stress on vulnerable workers [4, 27]. It also aligns with the publication of the International Labour Organization’s (ILO) *Guidelines for a Just Transition Towards Environmentally Sustainable Economies and Societies for All* [28], which represents an important policy landmark in identifying the need for occupational health and safety and social protection of workers in the face of climate change. A search strategy combining terms for *heat*, *health*, and *migrant worker* was developed by L.V and refined with support from K.L. and the university librarian. Previous reviews on similar topics were also used to inform the development of the strategy [9, 13]. The full search strategy can be found in Additional file 2: Table S1. In addition, we also searched key grey literature websites including the ILO, the International Organization for Migration (IOM), and the National Institute for Occupational Safety and Health (NIOSH). Relevant reports published by these institutes were searched for key references not picked up by the search strategy.

### Inclusion and exclusion criteria

Inclusion and exclusion criteria were developed using a population, exposure, and outcome framework, adapted from the population, intervention, comparison, outcome, and study design (PICOS) framework [29]. The population included international migrant workers, defined as individuals who are or have been employed outside their country of origin. The exposure of interest was occupational heat. Due to the inconsistent use of heat exposure indicators [21] and the varying levels at which individuals may experience heat stress [30], heat exposure is often difficult to define, so this criterion was reflected in the search strategy. Outcomes of interest included poor health outcomes (defined as impaired health or well-being of the workers), and interventions aimed at mitigating the impact of occupational heat on the health of migrant workers. Any comparators were included (e.g., host populations, migrant workers not exposed to occupational heat). We included all primary study designs, including cross-sectional, prospective cohort, and case-control studies, using both qualitative and quantitative approaches. Studies were excluded if they did not include migrant populations, if environmental temperatures made no contribution to high working temperatures, or if the study did not measure exposure during working hours. Language was restricted to English, but no geographical restriction was applied.

### Study selection procedure

Records obtained from the searches were imported into EndNote version 21 [31] to remove duplicates and then exported to the web-based application Rayyan [32] where remaining duplicates were removed. Titles and abstracts were screened by L.V; full-text articles included at this stage were retrieved and screened for eligibility by L.H; 25% of the screening process was duplicated by M.J. Disagreements at any stage were resolved through consensus.

### Data extraction

The information extracted included author, publication date, study design (e.g., observational, experimental), country of study, study setting (e.g., urban, rural), and period. Population characteristics included occupation, industry, country of origin, length of stay, age range, and gender. Exposure characteristics involved a measure or description of heat exposure. Outcome characteristics included a description or diagnosis of the health problem. Interventions were defined as any action implemented by study participants, employers, or members of the research team aimed at mitigating poor health outcomes caused by exposure to high ambient temperatures. Information extracted about interventions included intervention characteristics (description of the intervention), positive outcomes, negative outcomes, and any documented impact. For studies with a majority migrant population, data referring to the whole population were used. For studies where migrants made up half or less of the population, only data regarding migrants were used. 50% of data extraction was duplicated by S.W, with studies selected at random. Disagreements were resolved through consensus.

### Quality assessment

We used the Joanna Briggs Institute (JBI) checklists most appropriate for the study designs of the included studies to assess the risk of bias [33, 34]. A scoring system was used, where studies scoring between 0 and 3 were considered low quality, those scoring between 4 and 6 were considered of average quality, and those scoring between 7 and 10 were considered high quality, as was done in a similar review [9]. Quality assessment was duplicated by M.J with disagreements resolved through consensus. Studies were not excluded based on quality.

### Data synthesis

The methodology of the results synthesis is that of narrative synthesis. The Economic and Social Research Council (ESRC) Methods Programme Guidance was used, paying particular attention to elements 2 and 3, *developing a preliminary synthesis* and *exploring relationships in the data*, respectively [35]. Data were collated, then tabulated and displayed to best summarise the findings. The data were separated into subgroups where appropriate and compared accordingly. Qualitative data such as protective interventions were manually analysed for trends and common themes.

## Results

### Overview of included studies

**Fig. 1:**
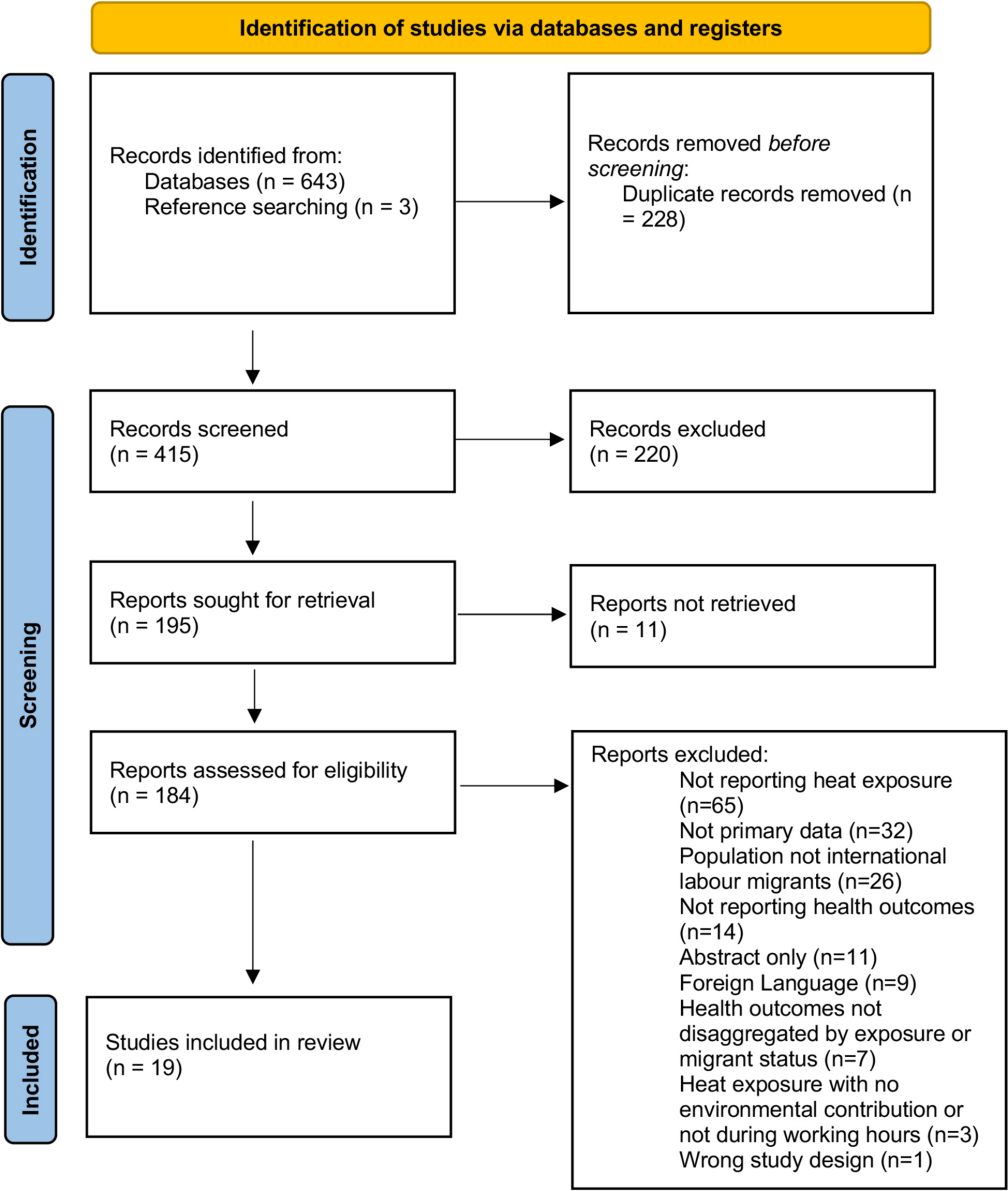
PRISMA flowchart of included studies

**Fig. 2:**
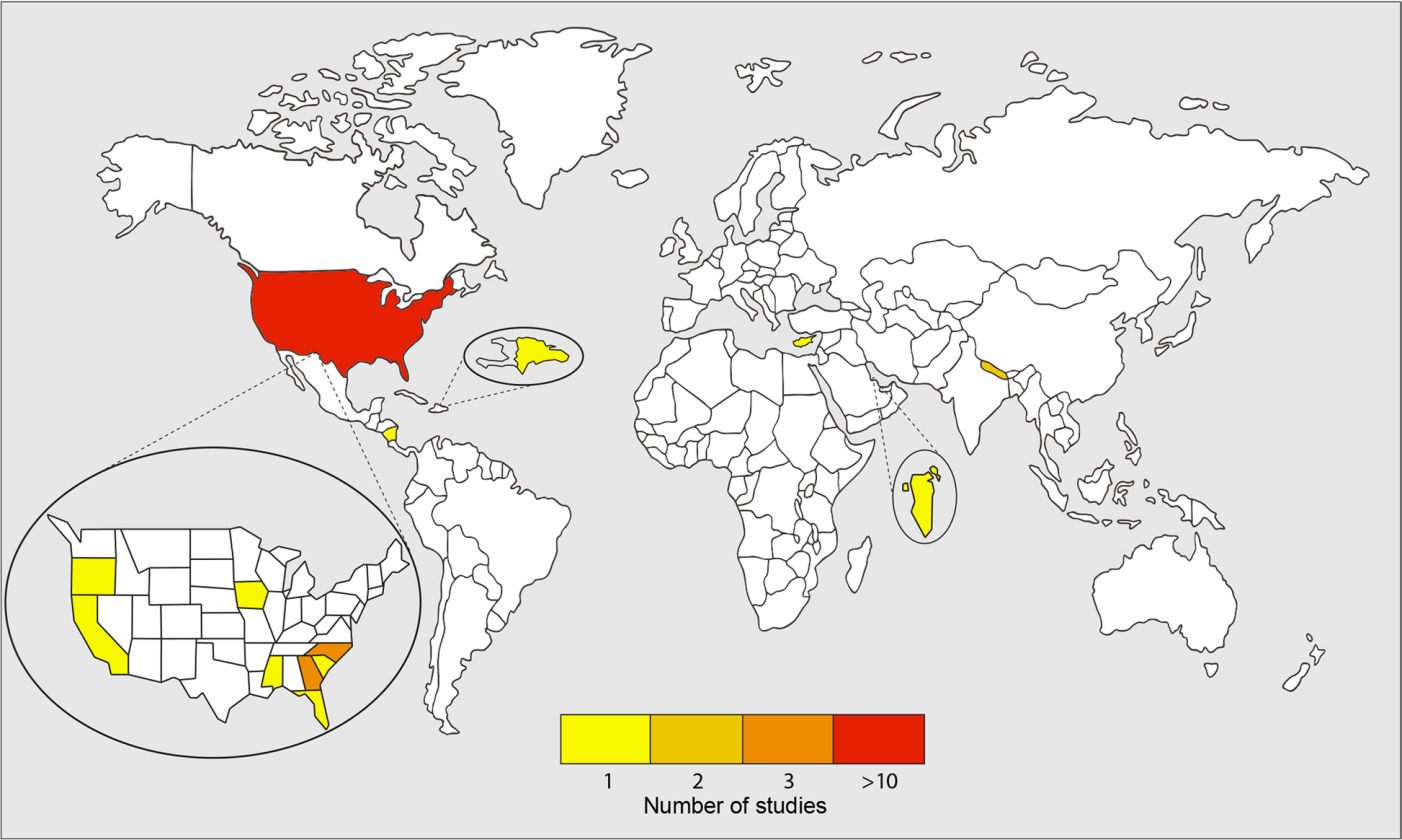
Global distribution of included studies. Figure shows that most studies are distributed in North America. Other studies are in Costa Rica (*n = *1) [50], the Dominican Republic (*n = *1) [46], Cyprus (*n = *1) [17], Bahrain (*n = *1) [37], and Nepal (*n = *2) [42, 52]. Within North America, 3 studies were conducted in Georgia [43, 44, 48], 3 in North Carolina [40, 41, 47], and 1 in each of Florida, Iowa, California, Mississippi, South Carolina, and Oregon [36, 38, 39, 45, 49, 51]

Of the 646 records screened, 195 full texts were assessed for eligibility, of which 19 studies involving 2293 migrant workers across six countries were included in the final analysis [17, 36–53] (see Fig. 1 PRISMA flowchart) (Table 1). Studies were mainly conducted in high-income countries (HICs) (*n = *14, 74%); the United States of America (USA) (*n = *13) and Bahrain (*n = *1) [37], with the remaining studies in low and middle-income settings: Nepal (*n = *2) [42, 52], Costa Rica (*n = *1) [50], Cyprus (*n = *1) [17], and the Dominican Republic (*n = *1) [46]. The studies in the USA were carried out in different states including North Carolina (*n = *3) [40, 41, 47], Georgia (*n = *3) [43, 44, 48], Florida (*n = *1) [36], Iowa (*n = *1) [38], Mississippi (*n = *1) [51], Oregon (*n = *1) [45], South Carolina (*n = *1) [49], California (*n = *1) [39], and one unspecified [53] (see Fig. 2). Study locations varied with three conducted at migrants’ places of work [17, 36, 38], ten in health care facilities [37, 42–44, 46, 47, 49, 51–53], five in worker accommodation or the community [40, 41, 45, 48, 50], and one unspecified [39]. All studies reporting the work environment described all or some work occurring outdoors. Indoor work was limited and included agricultural work in packing sheds [48], 43% of construction workers who work partly or fully indoors [37], and workers in housekeeping, restaurant work and factory work in another study [44]. The majority of studies had a cross-sectional design (*N = *9) [53–61] Nine studies were found to be of low quality [37, 41, 42, 46, 48, 50–53], eight of average quality [17, 36, 38, 39, 43–45, 49], and two of high quality [40, 47] (see Additional file 2: Table S2, S3, and S4).

**Table 1:** General characteristics of included studies

Study	Study period	Location	Setting	Population	Migrant population (*N*)	Study focus	Quality score
Abasilim et al. [36]	2021–2022	Florida, USA	Vegetable farm	Migrant farmworkers	111	Dehydration	6
Al-Sayyad and Hamadeh [37]	2008	Bahrain	Workers’ health centre	Labourers	1111	Climate-related health conditions	3
Arnold et al. [40]	2017	North Carolina, USA	The community	Latinx child farmworkers	30	Heat-related illness	7
Culp and Tonelli [38]	2019	Iowa, USA	Farms	Hispanic Farmworkers	155	Heat-related illness	4
Crowe et al. [50]	2011	Costa Rica	Sugarcane farm labour camp	Sugarcane harvesters	91	Heat-related symptoms	3
Ioannou et al. [17]	2016–2019	Cyprus	Agricultural farms	Agricultural workers	92	Occupational heat strain risk	5
Kearney et al. [41]	2014	North Carolina, USA	Migrant labour camps, housing and barracks	Latino migrant farmworkers	157	Sun protection behaviors	5
Keeney et al. [39]	2021	California, USA	Not specified	Latina farmworkers	60	Work-life stress	6
Luque et al. [49]	2017	South Carolina, USA	Clinics; a migrant head start facility	Hispanic farmworkers	29	HRI knowledge, attitudes, perceptions and beliefs	6
Luque et al. [48]	2018	Georgia, USA	Farmworker housing units	Hispanic farmworkers	99	Heat-safety knowledge, prevention, HRI risk perception	3
Madaras et al. [53]	2019	USA	Community health clinic	Migrant farmworker	1	Health care, social distance, and mobility	2
Mizelle et al. [47]	2020	North Carolina, USA	Health centre	Latino farmworkers	30	Fluid intake and hydration status	7
O'Connor et al. [46]	2017	Dominican Republic	Mobile clinic	Dominican batey communities	41	Foot health	3
Pokhrel et al. [52]	2018	Nepal	Hospital obstetrics and gynaecology department	Male partners of infertile couples	86	Infertility risk factors and semen abnormality	3
Sharma et al. [42]	2023	Nepal	Haemodialysis centres	Endstage renal disease patients	95	Environmental and occupational exposures	2
Smith et al. [43]	2018	Georgia, USA	Mobile worksite clinic	Migrant farmworkers	60	Knowledge of HRI first aid	5
Smith et al. [44]	2019	Georgia, USA	Hospital emergency haemodialysis services	Undocumented workers	50	Occupational exposures	4
Stoklosa et al. [51]	2020	Mississippi, USA	Hospital emergency department	Migrant agricultural worker	1	Pesticide exposure, heat exhaustion, labour trafficking	3
Wilmsen et al. [45]	2019	Oregon, USA	Forestry services network	Latino forest workers	23	Occupational injuries	5

Regarding the social characteristics of migrant workers, ages ranged from 10 to 90 years old, Of studies reporting information on sex (*n = *1282 participants), about 76% were male (*N = *973) versus 24% female (*N = *309). Countries of origin included India (*n = *865), Mexico (*n = *573), Nepal (*n = *227), Bangladesh (*n = *100), Nicaragua (*n = *91), Pakistan (*n = *78), Haiti (*n = *41), Romania (*n = *40), Guatemala (*n = *19), Vietnam (*n = *18), Philippines (*n = *12), El Salvador (*n = *5), Bulgaria (*n = *3), and Honduras (*n = *3) [17, 36, 37, 39–46, 48–53]. Work sectors included construction (*N = *1113, 48%) and agriculture (crop production) (*N = *971, 42%), with fewer studies including migrants in services (e.g., cleaning and restaurant work) (*N = *30, 1%), forestry (*N = *23, 1%), and manufacturing (*N = *14, 1%).

### Heat exposure and related health outcomes

Heat exposures were described in various ways. Temperatures were reported as wet bulb globe temperature (WBGT) [17, 36, 47, 50], ambient temperature [48, 49], and heat index [43]. WBGT has a range of 18.7 °C to 32.5 °C [17, 36], and ambient temperature of 33.3 °C to 43.3 °C [49, 51]. Non-numerical descriptions of heat exposure included the words *hot* and *humid*– often together, *high temperatures*, and *occupational heat exposure* [38–40, 42, 44, 49, 51, 52].

**Fig. 3:**
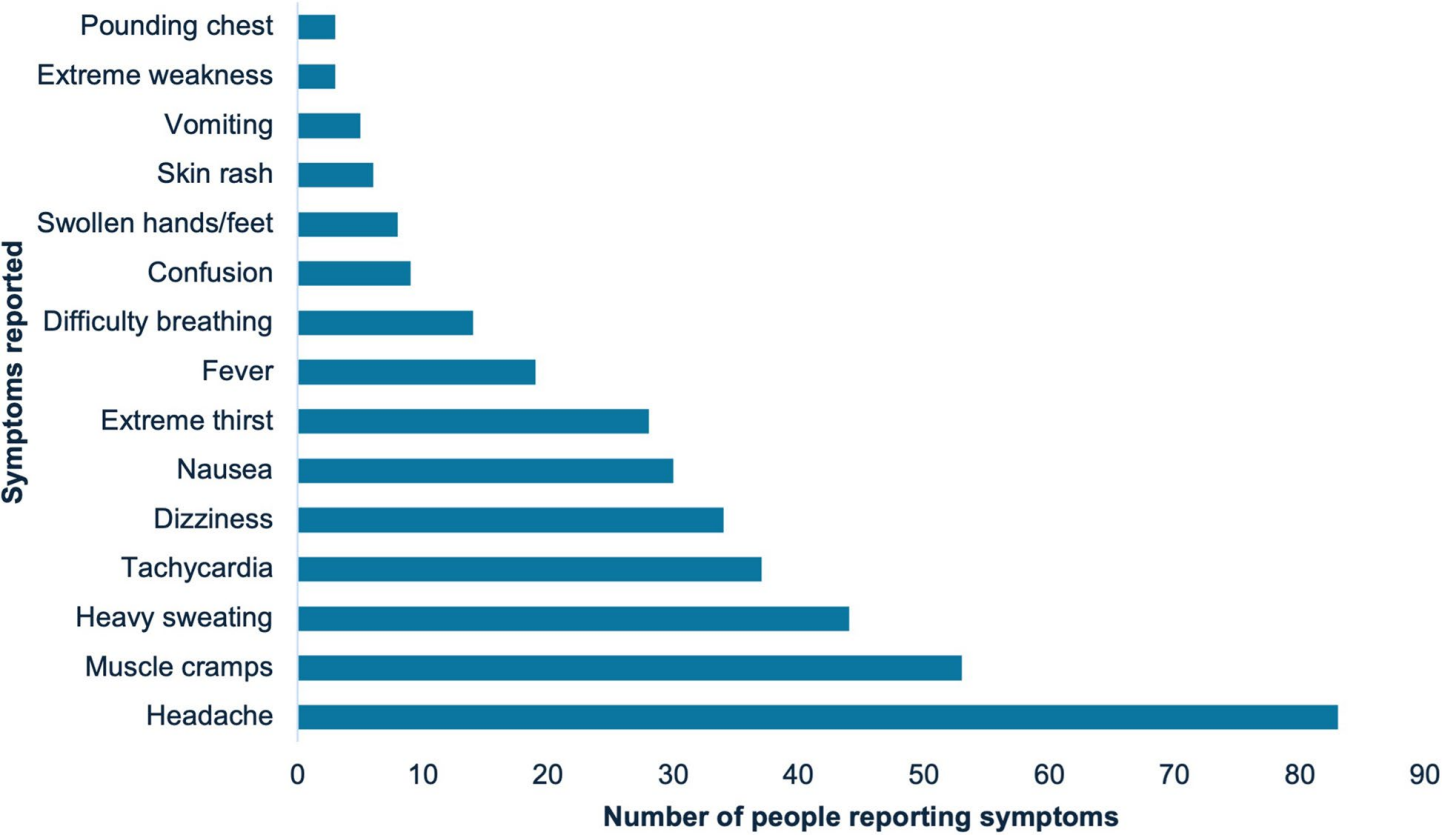
Prevalence of heat-related illness symptoms reported in included studies. Figure shows the most and less common symptoms of heat-related illness reported within 7 included studies (36, 38, 43, 48–51)

A range of poor health outcomes related to occupational heat exposure were reported (Table 2). Heat strain was the most commonly reported (*n = *6) [17, 36, 38, 43, 48, 49], followed by dehydration (*n = *5) [36, 47, 49–51]. Other reported outcomes included infertility [52], kidney disease [42, 44, 51] and compromised skin health (including dry skin and sun damage) [41, 46]. One study identified a risk to pregnancy as a poor health outcome [53]. Two studies also reported mental health outcomes [36, 39]. Heat-related illness (HRI) was reported in three studies with no further specification or clear definition [37, 40, 45], using diagnoses of heat strain or heat exhaustion [17, 42, 50, 51], or through symptomatology [36, 38, 43, 48, 49]. Five studies had sufficient data on symptoms of HRI to be collated (Fig. 3) [38, 43, 48, 50, 51]. The most common symptoms among migrant workers related to HRI were headache (*n = *83), muscle cramps (*n = *53), heavy sweating (*n = *44), tachycardia (*n = *37), and dizziness (*n = *34). Additional symptoms experienced by workers were nausea (*n = *30), extreme thirst (*n = *28), fever (*n = *19), difficulty breathing (*n = *14), confusion (*n = *9), swollen hands or feet (*n = *8), skin rash (*n = *6), vomiting (*n = *5), extreme weakness (*n = *3), and pounding chest (*n = *3). For symptoms reported without prevalence data, a minimum of one person reporting it was assumed [36, 38, 49]. Symptoms reported only once in total included heavy breathing, simultaneous hot and cold feeling, heart palpitations, dry skin, muscle spasms, stomach cramps, and weakness [38, 48, 49].

**Table 2:** Summary of reported health outcomes in the included studies

Study	Health outcome	Main symptoms and/or signs^1^	Population affected	Related exposure (°C)
Abasilim et al. [36]	Dehydration	USG (mean start, middle, end of shift) 1.022, 1.025, 1.029	Migrant farmworkers	WBGT (mean start, middle, end of shift): 18.7, 21.8, 24.7
Crowe et al. [50]		Dry mouth, dysuria	Sugarcane harvesters	WBGT > 26 for majority of shift
Luque et al. [49]		None	Hispanic migrant farmworkers	*Hot and humid*, temperature between 37.8 and 43.3
Mizelle et al. [47]		None	Migrant farmworkers	WBGT: mean 29.1, mean maximum 33.9
Stoklosa et al. [51]		None	Migrant agricultural worker	*Hot and humid*, temperature 33.3
Crowe et al. [50]	Heat exhaustion	Headache, tachycardia, muscle cramps	Sugarcane harvesters	WBGT > 26
Sharma et al. [42]		None	Returnee migrant workers	Occupational heat exposure, daily in 65%
Stoklosa et al. [51]		Light-headedness and syncope	Migrant agricultural worker	*Hot and humid*, temperature 33.3
Al-Sayyad and Hamadeh [37]	Heat-related illness (unspecified)	*Heat-related disease*	Construction labourers	Not described
Wilmsen et al. [45]		*Heat illness*	Latino forest workers	Not described
Arnold et al. [40]		HRI symptoms	Latinx child farmworkers	*Extremely hot*
Abasilim et al. [36] Ioannou et al. [17]	Heat strain	Headache, cramps, and dizziness	Migrant farmworkers	WBGT (mean start, middle, end of shift): 18.7, 21.8, 24.7
Culp and Tonelli [38]		Extreme thirst, muscle cramps, confusion	Hispanic farmworkers	*Hot and humid*
Luque et al. [49]		Various^2^	Hispanic migrant farmworkers	*Hot and humid*, temperature between 37.8 and 43.3
Ioannou et al. [17]		Between 17% and 27.7% of work shift spent above Tc 38 °C, heat strain diagnosed	Agricultural workers	Average WBGT: 24.8, highest: 32.5
Luque et al. [48]		Headache, heavy sweating, skin rash	Hispanic farmworkers	Average high temperature 33.5
Smith et al. [43]		Heavy sweating, cramps, headache	Migrant farmworkers	Maximum daily heat index: 29.1, relative humidity 85.5%
Pokhrel et al. [52]	Infertility	Abnormal semen parameters	Migrant Gulf country workers	*Heat exposure*
Smith et al. [44]	Kidney disease	None	Undocumented migrant workers	*Occupational heat exposure*
Stoklosa et al. [51]		Renal insufficiency, elevated creatinine kinase	Migrant agricultural worker	*Hot and humid*, temperature 33.3
Sharma et al. [42]		End-stage renal disease	Returnee migrant workers	Occupational heat exposure, daily in 65%
Kearney et al. [41]	Poor skin health	Sunburn, premature photoaging, skin cancer	Latino migrant farmworkers	High level of sun exposure, 0–5 h per day (*n = *3), 6–8 h (*n = *16), and ≥ 9 h (*n = *135)
O'Connor et al. [46]		Dry skin (xerosis)	Dominican Batey inhabitants	Constant exposure to high air temperatures and sun
Abasilim et al. [36]	Poor wellbeing	Wellbeing bad or very bad	Migrant farmworkers	WBGT (mean start, middle, end of shift): 18.7, 21.8, 24.7
Keeney et al. [39]		Stress	Latina migrant farmworkers	*Occupational heat exposure*
Madaras et al. [53]	Risk to pregnancy	none	Migrant farmworker	*Occupational heat exposure*

*USG *urine specific gravity, *WBGT *wet bulb globe temperature, *HRI* heat-related illness, *Tc* core body temperature

^1^Where more than three were reported, the three most prevalent have been included

^2^Prevalence not recorded. Symptoms described were breathing heavily, dehydration, feeling sick, headache, combined hot and cold body temperatures, excessive sweating, rashes, nosebleeds, bloodshot eyes, inability to move body (participant had recently undergone chemotherapy), heart palpitations, dizziness, and muscle spasms

### Protective interventions and strategies to mitigate the impact of occupational heat

Twelve studies described interventions and strategies to mitigate heat-related poor health outcomes in migrant workers (Table 3). Interventions included those related to water (*n = *4 studies) [36, 38, 47, 49], skin protection (*n = *3) [38, 41, 48], education (*n = *3) [47–49], shade (*n = *2) [48, 49], healthcare services (*n = *2) [38, 53], work environment (*n = *1) [45], rest (*n = *1) [38], and international guidelines (*n = *1) [47]. Water-related interventions, including the availability of water in the workplace, were recorded, yet challenges persisted. Where water, drinking vessels, and time to drink were provided, dehydration still worsened throughout shifts and workweeks [36]. When cold water was supplied, workers reported unpleasant symptoms like muscle spasms and lung cramps, and coolers were reportedly misused to store alcohol [49]. Despite adherence to National Institute for Occupational Safety and Health (NIOSH) hydration standards, workers reported being inadequately hydrated, with stomach pain from bending during work further discouraging fluid intake [47]. Protective measures related to rest time were reportedly met with resistance from vulnerable workers who refused to ask for breaks [38]—a problem commonly identified as a consequence of payment structures that incentivize workers to keep working [54]. While some workers were reported to seek shade to reduce the impact of OHS, safe shaded areas were scarce, sometimes leading to unsafe practices like resting under trucks [48]. Skin protection strategies, such as wearing long-sleeved clothing, were reported to be effective in shielding workers from solar radiation [38], but in the case of headgear, the most protective style interfered with work tasks, leaving areas like the neck and face exposed when unworn [41].

**Table 3:** Summary of protective interventions, strategies, and their documented impact

Intervention	Description	Outcome
Water	Drinking vessel, cold water and hydration period provided.	25.1% dehydrated start of shift, 82.7% end of shift. Progressive dehydration throughout workweek [36].
	Employers encouraged fluid intake.	Supervisors not tracking or reminding individuals to drink [38].
	Coolers stocked with water supplied.	Drinking cold water associated with muscle spasms and lung cramps. Crew leaders would stock the cooler with beers during longer shifts [49].
	29/30 drinking water every 15–20 min (and therefore meeting NIOSH standards).	Rehydration before and after work potentially inadequate; dehydration prevalent. Stomach pain when bending over to harvest following drinking water prevented workers from drinking more [47].
Rest	Researchers recommended that vulnerable individuals ask permission to rest.	Individuals refused [38].
Shade	62% took breaks in shade.	Limited access to shade under trees, trucks used instead [48].
	Workers took shade under trucks.	Compromised safety and further exposure to heat [49].
Skin protection	Long sleeved shirts worn by workers.	Protected from solar radiation and sunburn [38].
	Workers wore long-sleeves, long pants, collared shirts, and hats.	Most protective wide-brimmed hat interfered with work leaving back of neck, ears, lips and some of face not protected from sun exposure [41].
	Most workers wore head to toe clothing as sun protection.	77% reported never/rarely using sunglasses [48].
Education	Training from the researchers on the Heat Safety Tool app.	Participants interested in learning more about weather information and HRI risks [49].
	Farmworkers understood the basic connection between water intake and kidney function.	Rehydration before and after work potentially inadequate, dehydration prevalent [47].
	Crew leaders trained on OSHA heat illness prevention (water, rest, shade) and given OSHA Heat Safety Tool App to download prior to harvest season.	32% of workers had received heat safety training. 81% of workers had a cell phone, 61% reported using apps; app was highly rated by crew leaders in all domains [48].
Work environment	Improving work safety climate.	Employers followed through with better medical care [45].
Healthcare services	On-site health clinics provided.	2.7% of workers used on-site clinics [38].
	Patient enrolled in a case-management system for mobile patients to maintain her care.	Patient’s continuity of care was maintained for post-partum whilst she moved for work every 3–6 weeks [53].
International guidelines	NIOSH guidance to avoid afternoon heat [62].	Workers exceed recommended limits for occupational heat exposure by 9.30am [47].

*NIOSH* National Institute for Occupational Safety and Health, *OSHA *Occupational Safety and Health Administration, *HRI* heat-related illness

Regarding educational interventions, workers expressed interest in learning about heat-related illness and its risks [49];however a knowledge-to-action gap remained as awareness of the links between hydration and kidney health did not translate into adequate hydration practices [47]. The Occupational Safety and Health Administration (OSHA) training and the Heat Safety Tool app were well-received by crew leaders, yet only 32% of workers reported receiving heat safety training despite 81% having access to mobile phones with which to utilise the app [48]. Improvements to the working environment, such as better work-safety parameters, led to better medical care provision [45], but healthcare services like on-site clinics had low utilization amongst workers, despite higher percentages of heat-related illness symptoms [38]. Language barriers further complicated access to care, as many workers lacked proficiency in the local language, limiting medical consultations and exacerbating work-life stressors [39, 45, 51]. Mobile case management systems, however, proved effective in maintaining continuity of care for a mobile worker [53]. International guidelines of NIOSH recommendations to avoid working in the afternoon heat, failed to prevent overexposure in practice. Workers in California were found to have exceeded recommended heat exposure limits by mid-morning during the summer months [47].

## Discussion

This systematic review identified heat-related health risks among 2293 migrant workers across six countries, with over 80% employed in construction and agricultural sectors. At-risk workers included those as young as 10 years old and up to 90 years old, both age categories that increase workers’ risk of morbidity and mortality in extreme temperatures, highlighting a burden of multiple vulnerabilities within this study population [55]. The study describes adverse health outcomes among migrant workers exposed to occupational heat, with the most commonly reported outcomes being heat strain and dehydration. Other reported health issues included kidney disease, infertility, compromised skin health (such as dry skin and sun damage), mental health effects, and risk to pregnancy. A range of symptoms related to heat-related illnesses were reported, with the most common being headaches, muscle cramps, heavy sweating, tachycardia, dizziness, nausea and extreme thirst. Despite these significant risks, we found limited research on health-related outcomes in migrant workers, particularly in LMICs. Various protective measures have been described, including water availability, rest breaks, shade, skin protection, education, workplace improvements, healthcare services, and some level of adherence to international safety guidelines. However, significant barriers persist, such as limited access to water and hydration breaks, reluctance to take rest periods—as a result of discriminatory payment structures, insufficient shaded and safe areas, practical challenges with protective clothing, gaps in translating knowledge into action, underutilization of healthcare services—in some cases due to language barriers, and poor implementation or inadequacy of safety guidelines.

The finding that most of those who are affected by heat stress work in construction and agriculture is in line with ILO calculations from 2019 that these two sectors would be the worst hit by reduced labour productivity resulting from heat stress [4]. The geographic spread of the studies included in this review shows that there is a high concentration of reporting in HICs, particularly in the USA, where workers are predominantly Mexican and Central American migrants. Migration from Mexico to the USA represents the largest migration corridor globally, and data on financial remittances sent by international migrant workers show that the sources of these are nearly always sent from HICs [3]. Consistent with our findings, a recent scoping review on OHS among outdoor migrant and ethnic minority workers found that most studies were conducted in the USA [23]. In this review, the weighted prevalence of experiencing at least one HRI symptom was estimated at 48.8%, while 27.7% experienced at least three symptoms with higher prevalence rates in studies outside the USA [23]. A single study in Bahrain reported a large population of migrant workers originating from India making it the largest group in this study [37]. Other countries across the Arabian Peninsula are host to large numbers of international migrant workers, particularly in the high-risk sectors of construction and agriculture, representing a geographic region where there may be limited reporting on occupational heat exposure in international migrant workers [2].

Despite the dearth of literature reporting from LMICs on this topic, trends in global exposure to extreme heat in relation to disease burden show that LMIC populations experienced a higher risk of exposure to extreme heat in 2010–2019 and a subsequent greater health loss than HICs [63]. Thus, research around heat and health in these countries is required to fill the knowledge gap regarding international migrant workers in LMICs and advise policy going forward. The effects of occupational heat exposure on migrant worker health, were identified amongst workers upon their return to their home countries, seen in the case of Nepal as found in this review [42, 52]. Other countries in the Asian subcontinent also receive a large amount of international remittances from emigrated workers [3], representing other countries in this region that may be receiving returnee migrant workers. Bangladesh, as well as Nepal, has been a focus for the reintegration of large numbers of returnee migrant workers [56, 64]. This indicates that protective interventions to mitigate occupational heat exposure could also be targeted towards international migrant workers upon their return to their home countries.

Our findings also indicate that analyses to compare evidence on heat-related illness had several limitations. Difficulties categorising HRI arose due to the inconsistent terminology used in the included studies. Diagnoses of heat-strain, heat-related disease, heat exhaustion, and heat illness were all used to describe experiences of HRI [17, 37, 42, 45, 50, 51]. To understand the burden of heat illness in this vulnerable population, more specific classification is required. Wight et al. [57] explain the process of public health intervention development, the first step being the clarification of the problem, emphasising the need for clear definitions of the health issue and its cause. Reporting of heat exposure is not standardised in the included literature and is often poorly defined. Temperatures were measured using different methods, with only a few studies using WBGT– the method recommended by OSHA for monitoring workplace heat levels [17, 36, 47, 50, 65]. Increased consistency in exposure and outcome reporting is needed for the creation of effective, targeted interventions for OHS and to enable cross-study comparisons. This issue has been reported by other researchers on similar topics [23], limiting such comparability.

The findings from the systematic review reveal progress and gaps in interventions and measures aimed at protecting migrant workers from heat exposure. While the OSHA-recommended framework of water, rest, and shade has informed many measures in this review, its implementation often falls short, as seen in cases where inadequate shade or misuse of resources, such as coolers stocked with alcoholic drinks, exacerbated risks [48, 49]. A combined intervention involving water, rest, and shade was seen to reduce symptoms of heat stress and dehydration in a cohort of non-migrant workers, suggesting that a more rigorous implementation of these guidelines in combination would be beneficial in populations of migrant workers [58]. Educational interventions have shown promise in improving safety knowledge [66], but their reach remains limited, with training and safety information sometimes failing to reach workers [38, 48]. Farmworkers expressed a desire for more information on heat risks [49], underscoring the need for worker-centered approaches, including direct education and involvement in designing safety measures. This demonstrates how systemic barriers such as weak enforcement of regulations and lack of employer accountability must be addressed to ensure effective and sustainable protections.

Shortcomings in hydration practices and healthcare accessibility for migrant workers exposed to heat are also highlighted by this review. NIOSH standards recommending water intake every 15–20 min were insufficient to prevent dehydration, which worsened despite the provision of water, drinking vessels, and designated breaks [36, 47]. Regular water intake alone failed to counteract dehydration, a risk factor for AKI [47, 59], which is also exacerbated by hyperthermia and physical labor [67]. Electrolyte drinks and oral rehydration solutions (ORS) have been identified as more effective in replenishing lost fluids and electrolytes, with ORS showing superior fluid retention during exertion [60, 68]. These findings suggest a need to investigate ORS as a targeted intervention for dehydration and AKI prevention in this population. Additionally, despite the availability of onsite clinics, usage remained low [38], and language barriers increased difficulties in healthcare access in other studies [39, 45, 51]. Language barriers, low income, lack of health insurance, and preferences for self-medication have been identified as key factors limiting healthcare access and utilization in other populations of migrant workers [69]. Further research across diverse settings is needed to explore barriers to healthcare access in work environments, and develop strategies to improve service utilization among international migrant workers.

We found that PPE designed to protect against sun exposure went unused because it interfered with workers’ tasks [41], and suggestions to take breaks were refused by vulnerable workers [38]. This example illustrates that interventions lacking acceptability by the target population might see low uptake and fail to achieve desired outcomes. In contrast, a backpack hydration system developed with farmworkers’ mobility in mind was widely accepted for use amongst migrant workers and resulted in increased water intake [61]. Similarly, a recent review showed that although regular breaks are allowed, some migrant and ethnic minority workers forgo them due to barriers such as the desire to earn more or fear of losing their jobs [23]. This demonstrates the value of involving workers in the design process of interventions to ensure their practicality and acceptability. Barriers to the uptake of these measures, such as piece-rate payment structures that disincentivize taking breaks, can then be identified and addressed.

In light of these findings, recommendations are focused on strengthening occupational heat exposure research amongst migrant workers in LMICs. Specific investigations should address barriers to occupational health services amongst migrant workers at risk of heat-related illness and the efficacy of ORS for improving hydration in working migrants. Going forward, heat illness prevention strategies should be enhanced using worker-targeted education and increased accessibility to weather monitoring and heat-safety information systems. Strategies to develop additional heat-protective interventions should prioritise participatory approaches engaging migrant workers.

This review has some limitations. One is the exclusion of articles in languages other than English. As this study was aiming to gain a global perspective on the impact of heat exposure on migrant workers, excluding those in a foreign language may have missed important findings in migrant populations affected by occupational heat exposure elsewhere, resulting in a distorted view of study reporting on this topic. Furthermore, for this reason, because there is limited reporting on this topic (particularly from LMICs and the Arabian Peninsula), and because of poor standardization of heat exposure and heat strain recording, these data are certainly an underestimation of the true prevalence and impact of heat stress in international migrant worker populations. Another limitation of this study is the large number of low-quality studies. Broad inclusion criteria regarding study quality were required to ensure all available data on this topic were investigated. The inclusion of studies assessed as low quality was deemed to be important due to the scoping nature of this review in a new and expanding field. For the same reason, this review does not identify health outcomes for which the confirmed cause is exposure to high environmental temperatures, only that which may be associated with such an exposure.

## Conclusions

This systematic review has identified a population of migrant workers worldwide whose health was adversely affected by occupational heat exposure. Interventions being implemented to mitigate against adverse health effects related to occupational heat exposure were identified. Unsuccessful outcomes from these interventions delineate the specific factors mediating poor health outcomes in migrant workers. Recommendations following these observations are that interventions must be migrant-focused, and interventions should be combined with heat-safety education of all workers and employers, which will improve their success and sustainability. These findings should inform the creation of policy and guidelines protecting migrant workers’ health. This review has found that, despite guidelines surrounding temperature limits, water drinking standards, and adaptive behaviours, migrant workers are still burdened by occupational heat exposure with associated health effects as severe as requiring hospitalisation. With the effects of global warming only intensifying, rapid correction of these shortfalls in protecting the health of migrant workers is required.

## Supplementary Information

Supplementary Material 1. PRISMA 2020 checklist

Supplementary Material 2: Table S1. Search strategy for databases Medline, Embase, Ovid Global Health and PsychINFO. Table S2. Quality appraisal results: JBI Checklist for Analytical Cross-Sectional studies. Table S3. Quality appraisal results: JBI Checklist for Qualitative Research. Table S4. JBI Checklist for Case Reports

## Data Availability

All data supporting the findings of this study are available within the paper and its Supplementary Information.

## References

[1] International Convention on the Protection of the Rights of All Migrant Workers and Members of Their Families, (July 2003, 1990).

[2] ILO global estimates on international migrant workers. international migrants in the labour force. Geneva: ILO; 2024.

[3] Marie McAuliffe LAO. Migration and migrants: a global overview. World Migr Rep. 2024;2024(1):e00034.

[4] Kjellstrom T, Maître N, Saget C, Otto M, Karimova T, editors. Working on a warmer planet: the impact of heat stress on labour productivity and decent work2019.

[5] Amo-Agyei S. The migrant pay gap: understanding wage differences between migrants and nationals. Geneva: ILO; 2020. p. xxviii, 164 (report) + 16 p. (executive summary) + [2] p. (briefing note).

[6] Ronda Pérez E, G. BF, Katia L, G. LJ, Emily F, Van Rossem R. Differences in working conditions and employment arrangements among migrant and non-migrant workers in Europe. Ethn Health. 2012;17(6):563–77. 23534504 10.1080/13557858.2012.730606

[7] Francavilla F, Lyon S, De Cock M. Profits and poverty: the economics of forced labour. 2nd edn ed. Geneva: ILO; 2024. p. vi, 36 p.

[8] Pega F, Govindaraj S, Tran NT. Health service use and health outcomes among international migrant workers compared with non-migrant workers: a systematic review and meta-analysis. PLoS One. 2021;16(6):e0252651. 34106987 10.1371/journal.pone.0252651 PMC8189512

[9] Hargreaves S, Rustage K, Nellums LB, McAlpine A, Pocock N, Devakumar D, et al. Occupational health outcomes among international migrant workers: a systematic review and meta-analysis. Lancet Glob Health. 2019;7(7):e872–82. 31122905 10.1016/S2214-109X(19)30204-9 PMC6565984

[10] Kwon O, Song JH, Kong JO, Ma SW, Lee YS, Ahn J. Occupational characteristics and health status of Vietnamese male migrant workers in the Republic of Korea. Saf Health Work. 2023;14(3):267–71. 37818215 10.1016/j.shaw.2023.08.001 PMC10562105

[11] Organization IL. Towards a fair deal for migrant workers in the global economy. Int Labour Rev. 2004;143(3):294–5.

[12] Popova N, Rakotonarivo A. ILO global estimates on international migrant workers - results and methodology - Third Edition. 2021. Contract No.: Report.

[13] Flouris AD, Dinas PC, Ioannou LG, Nybo L, Havenith G, Kenny GP, et al. Workers’ health and productivity under occupational heat strain: a systematic review and meta-analysis. Lancet Planet Health. 2018;2(12):e521–31. 30526938 10.1016/S2542-5196(18)30237-7

[14] Organization IL. Best practices in the GCC region and beyond. In: Qatar ILOPOftSo, editor. Doha: ILO; 2024.

[15] Flouris AD, Ioannou L, Dinas P, Mantzios K, Gkiata P, Gkikas G, et al. Assessment of occupational heat strain and mitigation strategies in Qatar. International Labour Organization, Doha, Qatar. 2019.

[16] Alahmad B, Shakarchi AF, Khraishah H, Alseaidan M, Gasana J, Al-Hemoud A, et al. Extreme temperatures and mortality in Kuwait: who is vulnerable? Sci Total Environ. 2020;732:139289. 32438154 10.1016/j.scitotenv.2020.139289

[17] Ioannou LG, Testa DJ, Tsoutsoubi L, Mantzios K, Gkikas G, Agaliotis G, et al. Migrants from low-income countries have higher heat-health risk profiles compared to native workers in agriculture. J Immigr Minor Health. 2023;25(4):816–23. 37208495 10.1007/s10903-023-01493-2 PMC10198783

[18] El Khayat M, Halwani DA, Hneiny L, Alameddine I, Haidar MA, Habib RR. Impacts of climate change and heat stress on farmworkers’ health: a scoping review. Front Public Health. 2022;10:782811. 35211437 10.3389/fpubh.2022.782811 PMC8861180

[19] Gasparrini A, Guo Y, Sera F, Vicedo-Cabrera AM, Huber V, Tong S, et al. Projections of temperature-related excess mortality under climate change scenarios. Lancet Planet Health. 2017;1(9):e360–7. 29276803 10.1016/S2542-5196(17)30156-0 PMC5729020

[20] Amoadu M, Ansah EW, Sarfo JO, Hormenu T. Impact of climate change and heat stress on workers’ health and productivity: a scoping review. The Journal of Climate Change and Health. 2023;12:100249.

[21] Levi M, Kjellstrom T, Baldasseroni A. Impact of climate change on occupational health and productivity: a systematic literature review focusing on workplace heat. Med Lav. 2018;109(3):163–79. 29943748 10.23749/mdl.v109i3.6851 PMC7689800

[22] Ioannou LG, Foster J, Morris NB, Piil JF, Havenith G, Mekjavic IB, et al. Occupational heat strain in outdoor workers: a comprehensive review and meta-analysis. Temperature. 2022;9(1):67–102. 10.1080/23328940.2022.2030634 PMC9154804 35655665

[23] van Selm L, Williams S, de’Donato F, Briones-Vozmediano E, Stratil J, Sroczynski G, et al. Occupational heat stress among migrant and ethnic minority outdoor workers: a scoping review. Curr Environ Health Rep. 2025;12(1):16. 40123011 10.1007/s40572-025-00481-y PMC11930879

[24] Nations U. Transforming our world: the 2030 Agenda for Sustainable Development. UN General Assembly; 2015. Contract No.: ARES/70/1.

[25] Organization WH. WHO global action plan on promoting the health of refugees and migrants, 2019–2030. 2024.

[26] Page MJ, McKenzie JE, Bossuyt PM, Boutron I, Hoffmann TC, Mulrow CD, et al. statement: an updated guideline for reporting systematic reviews. BMJ. 2020;2021:372. 10.1136/bmj.n71 PMC8005924 33782057

[27] Lee H, Calvin K, Dasgupta D, Krinmer G, Mukherji A, Thorne P, et al. Synthesis report of the IPCC Sixth Assessment Report (AR6). IPCC. 2023.

[28] Organization IL. Guidelines for a just transition towards environmentally sustainable economies and societies for all. ILO Geneva; 2015.

[29] Richardson WS, Wilson MC, Nishikawa J, Hayward RS. The well-built clinical question: a key to evidence-based decisions. ACP J Club. 1995;123(3):A12-3. 7582737 10.7326/ACPJC-1995-123-3-A12

[30] European Agency for S, Health at W. Heat at work – guidance for workplaces | safety and health at work EU-OSHA. 2023. Contract No.: Report.

[31] The EndNote Team. EndNote. EndNote 2025 ed. Philadelphia, PA: Clarivate; 2013.

[32] Ouzzani M, Hammady H, Fedorowicz Z, Elmagarmid A. Rayyan—a web and mobile app for systematic reviews. Syst Rev. 2016;5:210. 27919275 10.1186/s13643-016-0384-4 PMC5139140

[33] Moola S, Munn Z, Sears K, Sfetcu R, Currie M, Lisy K, et al. Conducting systematic reviews of association (etiology): the Joanna Briggs Institute’s approach. Int J Evid Based Healthc. 2015;13(3):163–9. 26262566 10.1097/XEB.0000000000000064

[34] Lockwood C, Munn Z, Porritt K. Qualitative research synthesis: methodological guidance for systematic reviewers utilizing meta-aggregation. Int J Evid Based Healthc. 2015;13(3):179–87. 26262565 10.1097/XEB.0000000000000062

[35] Popay J, Roberts H, Sowden A, Petticrew M, Arai L, Rodgers M, et al. Guidance on the conduct of narrative synthesis in systematic reviews. A Prod ESRC Methods Programme Vers. 2006;1(1):b92.

[36] Abasilim C, Friedman LS, Martin MC, Madigan D, Perez J, Morera M, et al. Risk factors associated with indicators of dehydration among migrant farmworkers. Environ Res. 2024;251(Pt 2):118633. 38462085 10.1016/j.envres.2024.118633 PMC12588689

[37] Al-Sayyad AS, Hamadeh RR. The burden of climate-related conditions among laborers at Al-Razi Health Centre, Bahrain. 2014.

[38] Culp K, Tonelli S. Heat-related illness in Midwestern Hispanic farmworkers: a descriptive analysis of hydration status and reported symptoms. Workplace Health Saf. 2019;67(4):168–78. 30724664 10.1177/2165079918813380 PMC7497869

[39] Keeney AJ, Quandt A, Flores D, Flores L Jr. Work-life stress during the coronavirus pandemic among latina farmworkers in a rural California region. Int J Environ Res Public Health. 2022;19(8):4928. 10.3390/ijerph19084928 PMC9028285 35457795

[40] Arnold TJ, Arcury TA, Sandberg JC, Quandt SA, Talton JW, Mora DC, et al. Heat-related illness among latinx child farmworkers in North Carolina: a mixed-methods study. New Solut. 2020;30(2):111–26. 32349618 10.1177/1048291120920571 PMC7363553

[41] Kearney GD, Phillips C, Allen DL, Hurtado GA, Hsia LL. Sun protection behaviors among Latino migrant farmworkers in eastern North Carolina. J Occup Environ Med. 2014;56(12):1325–31. 25479305 10.1097/JOM.0000000000000275

[42] Sharma S, Koirala S, Inagaki Y, Kafle RK, Koirala S, Khadka N, et al. WCN24-2472 environmental and occupational exposure among endstage renal disease patients in Kathmandu, Nepal. Kidney Int Rep. 2024;9(4):S576.

[43] Smith DJ, Ferranti EP, Hertzberg VS, Mac V. Knowledge of heat-related illness first aid and self-reported hydration and heat-related illness symptoms in migrant farmworkers. Workplace Health Saf. 2021;69(1):15–21. 10.1177/2165079920934478 32723031

[44] Smith DJ, Mac V, Thompson LM, Plantinga L, Kasper L, Hertzberg VS. Using occupational histories to assess heat exposure in undocumented workers receiving emergent renal dialysis in Georgia. Workplace Health Saf. 2022;70(5):251–8. 35112607 10.1177/21650799211060695

[45] Wilmsen C, Castro AB, Bush D, Harrington MJ. System failure: work organization and injury outcomes among Latino forest workers. J Agromedicine. 2019;24(2):186–96. 30734660 10.1080/1059924X.2019.1567421 PMC6476664

[46] O’Connor JJ, Enriquez M, Wipke-Tevis DD. Foot health assessment and problem identification in a Dominican batey community: a descriptive study. J Wound Ostomy Continence Nurs. 2020;47(4):397–402. 33290018 10.1097/WON.0000000000000664 PMC7727270

[47] Mizelle E, Larson KL, Bolin LP, Kearney GD. Fluid intake and hydration status among North Carolina farmworkers: a mixed methods study. Workplace Health Saf. 2022;70(12):532–41. 36002982 10.1177/21650799221117273

[48] Luque JS, Becker A, Bossak BH, Grzywacz JG, Tovar-Aguilar JA, Guo Y. Knowledge and practices to avoid heat-related illness among Hispanic farmworkers along the Florida-Georgia line. J Agromed. 2020;25(2):190–200. 10.1080/1059924X.2019.1670312 PMC7075471 31544652

[49] Luque JS, Bossak BH, Davila CB, Tovar-Aguilar JA. I think the temperature was 110 degrees!": work safety discussions among hispanic farmworkers. J Agromedicine. 2019;24(1):15–25. 30317928 10.1080/1059924X.2018.1536572 PMC7045709

[50] Crowe J, Nilsson M, Kjellstrom T, Wesseling C. Heat-related symptoms in sugarcane harvesters. Am J Ind Med. 2015;58(5):541–8. 25851165 10.1002/ajim.22450

[51] Stoklosa H, Kunzler N, Ma ZB, Luna JCJ, de Vedia GM, Erickson TB. Pesticide exposure and heat exhaustion in a migrant agricultural worker: a case of labor trafficking. Ann Emerg Med. 2020;76(2):215–8. 32362432 10.1016/j.annemergmed.2020.03.007

[52] Pokhrel S, Ghimire A, Chhetry M, Lamichane S, Shreewastav RK. Selected risk factors and pattern of semen abnormality in male partners of infertile couples in Eastern Nepal: a descriptive cross-sectional study. JNMA J Nepal Med Assoc. 2020;58(229):668–71. 33068088 10.31729/jnma.4882 PMC7580322

[53] Madaras L, Stonington S, Seda CH, Garcia D, Zuroweste E. Social distance and mobility - a 39-year-old pregnant migrant farmworker. N Engl J Med. 2019;380(12):1093–6. 30893530 10.1056/NEJMp1811501

[54] Pan Q, Sumner DA, Mitchell DC, Schenker M. Compensation incentives and heat exposure affect farm worker effort. PLoS One. 2021;16(11):e0259459. 34727122 10.1371/journal.pone.0259459 PMC8562852

[55] Arsad FS, Hod R, Ahmad N, Ismail R, Mohamed N, Baharom M, et al. The impact of heatwaves on mortality and morbidity and the associated vulnerability factors: a systematic review. Int J Environ Res Public Health. 2022;19(23):16356. 36498428 10.3390/ijerph192316356 PMC9738283

[56] Socio-Economic Reintegration of Returnee Migrant Workers of Bangladesh. 2024.

[57] Wight D, Wimbush E, Jepson R, Doi L. Six steps in quality intervention development (6SQuID). J Epidemiol Community Health. 2016;70(5):520. 26573236 10.1136/jech-2015-205952 PMC4853546

[58] Bodin T, García-Trabanino R, Weiss I, Jarquín E, Glaser J, Jakobsson K, et al. Intervention to reduce heat stress and improve efficiency among sugarcane workers in El Salvador: phase 1. Occup Environ Med. 2016;73(6):409–16. 27073211 10.1136/oemed-2016-103555 PMC4893112

[59] Roncal-Jimenez C, Lanaspa MA, Jensen T, Sanchez-Lozada LG, Johnson RJ. Mechanisms by which dehydration may lead to chronic kidney disease. Ann Nutr Metab. 2015;66(Suppl 3):10–3. 26088040 10.1159/000381239

[60] Bates GP, Miller VS. Sweat rate and sodium loss during work in the heat. J Occup Med Toxicol. 2008;3:4. 18226265 10.1186/1745-6673-3-4 PMC2267797

[61] Mizelle E, Modly LA, Smith DJ. Farmworker acceptability of backpack hydration systems. J Agromedicine. 2024;29(3):477–85. 38704610 10.1080/1059924X.2024.2349022 PMC11160487

[62] Coco A, Jacklitsch B, Williams J, Kim J-H, Musolin K, Turner N. Criteria for a recommended standard: occupational exposure to heat and hot environments. DHHS (NIOSH) Publication. 2016.

[63] Du Y, Jing M, Lu C, Zong J, Wang L, Wang Q. Global population exposure to extreme temperatures and disease burden. Int J Environ Res Public Health. 2022;19(20):13288. 36293869 10.3390/ijerph192013288 PMC9603138

[64] Profiling Returnee Migrant Workers for Labour Market Integration: IOM, Geneva; 2022.

[65] Administration OSaH. Prevention - Heat Hazard Recognition Occupational Safety and Health Administration: U.S. Department of Labor; [Available from: https://www.osha.gov/heat-exposure/hazards].

[66] Lara M, Díaz Fuentes C, Calderón J, Geschwind S, Tarver M, Han B. Pilot of a community health worker video intervention for immigrant day laborers at occupational health risk. Front Public Health. 2021;9:662439. 34368045 10.3389/fpubh.2021.662439 PMC8339200

[67] Chapman CL, Johnson BD, Vargas NT, Hostler D, Parker MD, Schlader ZJ. Both hyperthermia and dehydration during physical work in the heat contribute to the risk of acute kidney injury. J Appl Physiol. 2020;128(4):715–28. 32078468 10.1152/japplphysiol.00787.2019 PMC7191500

[68] Fan PW, Burns SF, Lee JKW. Efficacy of ingesting an oral rehydration solution after exercise on fluid balance and endurance performance. Nutrients. 2020;12(12):3826. 33333771 10.3390/nu12123826 PMC7765193

[69] Liu L, Gjebrea O, Ali FMH, Atun R. Determinants of healthcare utilisation by migrant workers in the state of Qatar. Health Policy. 2020;124(8):873–80. 32532567 10.1016/j.healthpol.2020.04.011

