# Supplementary material for: International migrant workers, heat exposure, and climate change: a systematic review of health risks and protective interventions
Source: BMC Glob Public Health. 2025 Dec 1;3:107. doi: 10.1186/s44263-025-00224-z (PMC12667051; doi:10.1186/s44263-025-00224-z)
Supplement: Supplementary file 2 — Supplementary Material 2: Table S1. Search strategy for databases Medline, Embase, Ovid Global Health and PsychINFO. Table S2. Quality appraisal results: JBI Checklist for Analytical Cross-Sectional studies. Table S3. Quality appraisal results: JBI Checklist for Qualitative Research. Table S4. JBI Checklist for Case Reports [file 44263_2025_224_MOESM2_ESM.docx]

**Table S1. Search strategy**

| **Search concept 1: Migrant workers** | |
| --- | --- |
| 1 | ((foreign* OR non-native* OR non-national* OR migrant* OR refugee* OR undocumented OR expat* OR traffick* OR immigrant*)adj3(work* OR labo?r OR labo?rer OR job OR staff OR occupation* OR employ* OR forestr* OR plantation* OR manufactur* OR construction OR farm* OR brick kiln OR factory OR sugarcane OR fisher* OR mining)) |
| 2 | economic adj2 (migrant OR immigrant) |
|  | **1 OR 2** |

**AND**

| **Search concept 2: Health outcomes** | |
| --- | --- |
| 1 | health OR injur* OR disease* OR hazard* OR exposure* OR accident* OR hygiene OR safety OR trauma* OR fatal* OR harm* OR death* OR ill OR illness* OR sick* OR syndrome* OR wound* OR fatigue OR risk* OR disabilit* OR morbidit* or mortalit* OR infect* OR disorder* OR condition OR pain OR pains OR sore* OR ache* OR unwell OR hospital* OR vulnerabilit* OR strain* OR stress* OR danger* OR well?being |
| 2 | respirat* OR musculoskeletal OR cardiovascular OR cancer* OR hypertensi* OR dermatitis OR allerg* OR fall* OR drown* OR exhaust* OR broken bone* OR toxic* OR burn* OR parasite* OR pesticide* OR insecticide* OR hydration OR asthma OR bronchitis OR pulmonary OR fatigue OR nephropathy* OR renal function OR urolithiasis OR kidney* OR ckd* OR aki OR anxi* OR depress* OR psychiatr* OR psycho*OR suicid* OR distress* |
| 3 | Dehydrat* OR exhaust* OR fatigue* OR syncope OR edema OR oedema OR HRI OR sunburn* OR cramp* OR rash OR miliaria OR stroke OR hyponatremia OR hypernatremia OR rhabdomyolysis |
|  | **1 OR 2 OR 3** |

**AND**

| **Search concept 3: Heat exposure** | |
| --- | --- |
| 1 | heat* OR warm* OR hot OR dry OR summer OR temperatures OR wbgt OR humid* OR arid OR ultraviolet OR UV OR drought OR thermal |
| 2 | sweat* OR hypertherm* OR strain OR electrolyte* OR tolerance |
| 3 | ((climate OR climatic)adj2(change OR event OR disaster OR crisis OR variation OR variabilit*)) OR extreme weather |
|  | **1 OR 2 OR 3** |

**Table S2. Quality appraisal results: analytical cross sectional studies**

|  | **Study:** | | | | | | | | | | | | |
| --- | --- | --- | --- | --- | --- | --- | --- | --- | --- | --- | --- | --- | --- |
| **Checklist item:** | **Abasilim et al. (36)** | **Al-Sayyad and Hamadeh (37)** | **Culp and Tonelli (38)** | **Crowe et al. (50)** | **Ioannou et al. (17)** | **Kearney et al. (41)** | **Luque et al. (48)** | **Mizelle et al. (47)** | **O'Connor et al. (46)** | **Pokhrel et al. (52)** | **Sharma et al. (42)** | **Smith et al. (43)** | **Smith et al. (44)** |
| Were the criteria for inclusion in the sample clearly defined? | No | Yes | Yes | No | No | Yes | No | Yes | No | Yes | Yes | Yes | Yes |
| Were the study subjects and the setting described in detail? | Yes | Yes | Yes | Yes | Yes | No | Yes | Yes | No | No | No | Yes | Yes |
| Was the exposure measured in a valid and reliable way? | Yes | No | No | Yes | No | No | No | Yes | Unclear | No | No | Yes | No |
| Were objective, standard criteria used for measurement of the condition? | Yes | Unclear | No | No | Yes | No | No | Yes | Yes | Yes | Unclear | No | Yes |
| Were confounding factors identified? | Yes | No | Yes | No | Yes | Yes | No | Yes | No | No | Unclear | No | No |
| Were strategies to deal with confounding factors stated? | No | No | No | No | No | No | No | No | No | No | Unclear | No | No |
| Were the outcomes measured in a valid and reliable way? | Yes | Unclear | No | No | Yes | No | Yes | Yes | Yes | Yes | Unclear | Yes | No |
| Was appropriate statistical analysis used? | Yes | Yes | Yes | Yes | Yes | Yes | Yes | Yes | Yes | No | Yes | Yes | Yes |
| Score: | 6 | 3 | 4 | 3 | 5 | 3 | 3 | 7 | 3 | 3 | 2 | 5 | 4 |
| Quality rating: | Average | Low | Average | Low | Average | Low | Low | High | Low | Low | Low | Average | Average |

Questions extracted from *JBI Critical Appraisal Checklist for Analytical Cross Sectional Studies* (33).

**Table S3. Quality appraisal results: qualitative research studies**

|  | **Study:** | | | |
| --- | --- | --- | --- | --- |
| **Checklist item:** | **Arnold et al. (40)** | **Keeney et al. (39)** | **Luque et al. (49)** | **Wilmsen et al. (45)** |
| Is there congruity between the stated philosophical perspective and the research methodology? | No | No | Unclear | No |
| Is there congruity between the research methodology and the research question or objectives? | Yes | Yes | Yes | Yes |
| Is there congruity between the research methodology and the methods used to collect data? | Yes | Yes | Yes | Yes |
| Is there congruity between the research methodology and the representation and analysis of data? | Yes | Yes | Yes | No |
| Is there congruity between the research methodology and the interpretation of results? | Yes | No | Yes | Yes |
| Is there a statement locating the researcher culturally or theoretically? | No | No | No | No |
| Is the influence of the researcher on the research, and vice- versa, addressed? | No | No | No | No |
| Are participants, and their voices, adequately represented? | Yes | Yes | Yes | Yes |
| Is the research ethical according to current criteria or, for recent studies, and is there evidence of ethical approval by an appropriate body? | Yes | Yes | No | No |
| Do the conclusions drawn in the research report flow from the analysis, or interpretation, of the data? | Yes | Yes | Yes | Yes |
| Score: | 7 | 6 | 6 | 5 |
| Quality rating: | High | Average | Average | Average |

Questions extracted from *JBI Critical Appraisal Checklist for Qualitative Research* (34).

**Table S4. Quality appraisal results: case reports**

|  | **Study:** | |
| --- | --- | --- |
| **Checklist item:** | **Madaras et al. (53)** | **Stoklosa et al. (51)** |
| Were patient’s demographic characteristics clearly described? | No | No |
| Was the patient’s history clearly described and presented as a timeline? | Yes | No |
| Was the current clinical condition of the patient on presentation clearly described? | No | No |
| Were diagnostic tests or assessment methods and the results clearly described? | No | Yes |
| Was the intervention(s) or treatment procedure(s) clearly described? | n/a | Yes |
| Was the post-intervention clinical condition clearly described? | n/a | No |
| Were adverse events (harms) or unanticipated events identified and described? | No | No |
| Does the case report provide takeaway lessons? | Yes | Yes |
| Score: | 2 | 3 |
| Quality rating: | Low | Low |

Questions extracted from *JBI Critical Appraisal Checklist for Case Reports* (33).
